# Defining the guiding principles of weight-inclusive nutrition education

**DOI:** 10.3389/fpubh.2026.1734480

**Published:** 2026-02-18

**Authors:** Jordan A. Levinson, Janet Gamble, Kelsey Rose, Bernice R. Garnett, Deborah Hinchey, Lizzy Pope

**Affiliations:** 1 Department of Nutrition and Food Sciences, University of Vermont, Burlington, VT, United States; 2 Department of Education, University of Vermont, Burlington, VT, United States; 3 Public Health Sciences, University of Vermont, Burlington, VT, United States

**Keywords:** diet culture, eating disorder prevention, nutrition education, nutrition science, weight inclusivity

## Abstract

Weight-centric nutrition education centers weight as a health indicator and teaches individuals methods for controlling their weight and shape. This approach has negative psychological and physiological consequences, including the development of eating disorder pathology. Alternatively, weight-inclusive nutrition education disrupts anti-fat bias, may act as primary prevention of eating disorders, and offers a more sustainable approach to wellbeing. Weight-inclusive nutrition has roots in established concepts such as Health at Every Size® and Intuitive Eating, as well as interdisciplinary public health and social science theories such as the social ecological model and social cognitive theory. This paper outlines the core principles of weight-inclusive nutrition education.

## Introduction

Historically, nutrition science has aligned with weight-centric beliefs, placing personal responsibility on an individual to control their weight, and using weight (or Body Mass Index) as an indicator of health. This is in line with a broader societal sense of anti-fat bias, defined as “the attitudes, behaviors, and social systems that specifically marginalize, exclude, underserve, and oppress fat bodies” (1). More specifically, nutrition educators, medical providers, and policy makers function under the assumption that making an individual aware that their weight is a problem will result in individual behavioral change through increased education about diet, exercise and self-regulatory skills dictated by external cues (2). Practices such as calorie counting, nutrition-label literacy, restriction or elimination of certain food items, the dichotomy of food as good or bad, and portion control are common in nutrition counseling or individual behavior change-based interventions. Nutrition policy makers often deploy policies designed to educate the population about food with a focus on weight control. For example, the addition of calorie labels on menus is a strong example of a weight-centric nutrition policy designed to educate the populace. While this practice is aimed at increasing awareness of the calorie count of foods, research shows that this type of menu labeling can contribute to or exacerbate disordered eating and eating disorder symptoms (3). Similarly, policies that dictate what foods can or cannot be served in specific settings, i.e., schools, hospitals, daycares, long-term care facilities etc., have also created structural contexts that reinforce weight-centric ideals about the morality of food.

Ironically, weight-centric nutrition approaches are ineffective at generating sustained, long-term weight loss (4–6) and can have serious negative consequences for mental and physical health. This focus on weight and weight loss can contribute to harmful behaviors, such as weight cycling leading to subsequent cardiometabolic disease (7), as well as eating-disorder pathology. Research shows that engaging in dieting predicts eating disorder development and that weight-related self-monitoring, a common nutrition intervention, increases eating disorder symptomology (8). Alternatively, weight-inclusive approaches to nutrition education do not see it as an individual’s personal responsibility to control their weight or use weight as a health indicator (9). Weight-inclusive nutrition (WIN) is a value-based implementation framework that seeks to disrupt anti-fat bias, uncouple the linear, often reductive, rhetoric around weight and health, and acknowledge social determinants of health. It attempts to challenge the dominant obesity-prevention paradigm that assumes that if an individual is educated about healthy eating and movement, behavior change will occur (10), often labeling anyone that cannot meet this goal as lazy, lacking will power, or content with being “unhealthy” (11). Given the negative impacts of weight-centric approaches (12), a paradigm shift to weight-inclusive nutrition education occurring in a variety of settings - both formally and informally - could, in itself, act as primary prevention of eating disorders (13–17).

Previous eating-disorder prevention programs have concentrated on fostering positive body image through focusing on factors that are known to contribute to eating-disorder pathology such as thin-ideal internalization (18) and negative affect (19). Interventions aimed at improving body image and preventing eating disorders often do not address fundamental nutrition principles. For example, several curricula aimed at improving body image have been released in recent years including BE REAL’s *BodyKind^TM^ and Let us Eat!*, Dove’s *Confident Me,* and the National Eating Disorders Association *Body Project.* Many of these curricula do not address the relationship that exists between body image and behavioral choices around food. *BodyKind*^TM^ and *Let us Eat!* are the only curricula to address nutrition in what is referred to as “tuned-in eating,” with an emphasis on recognizing hunger and fullness cues and a focus on gentle nutrition, or choosing to eat foods that honor one’s well-being, wants, culture, and hunger (20). Yet, many of the other core weight-inclusive nutrition principles that we propose below are not explicitly addressed in these curricula, emphasizing that current programing on body image remains separate to weight-inclusive nutrition education. Further, while fostering a positive relationship to body is essential for overall wellbeing and eating-disorder prevention (21), it presents only one part of a healthy relationship to food and body. Nutrition knowledge is essential to fostering this relationship and promoting both physical and mental wellbeing.

It is important to adopt a weight-inclusive mindset in settings in which the traditional, primary approach to nutrition is weight centric. Previous literature describes the need for and tenets of weight-inclusive *healthcare* (9, 12), characterized by the belief that health can be more safely and sustainably pursued independent of weight, and that individuals of all body sizes deserve quality, non-stigmatizing medical care. However, to our knowledge, no one has defined the principles of *nutrition* from a weight-inclusive perspective. In this paper, we aim to describe how weight-inclusive nutrition differs from weight inclusivity in general, outline the core principles of weight-inclusive nutrition which can be adapted to a variety of formal (e.g., dietetics education) and informal (e.g., nutrition counseling or public health campaigns) educational settings, and describe how weight-inclusive nutrition can be applied in various settings.

## The guiding principles of weight-inclusive nutrition (win)

When creating the weight-inclusive nutrition guiding principles we aimed to merge scholarship around weight-inclusivity (22) with scholarship around anti-diet approaches such as intuitive eating (20) and Health at Every Size® (23) with the overarching goal of providing nutrition education in a way that would foster more positive relationships with food, and not catalyze the development of disordered-eating behaviors or contribute to anti-fat bias. Furthermore, the guiding principles needed to include basic information about nutrition science while also helping people apply that science to their individual eating goals. Subtopics within the guiding principles either reflect basic nutrition concepts and skills or are important to discuss in terms of their connection to disordered eating, eating disorders, weight cycling, or anti-fat bias.

The five guiding principles that define WIN are (1) Centers a foundation of nutrition science (2) Analyzes the relationship between nutrition and health outcomes, (3) Examines nutrition information critically, (4) Promotes attuned and joyful eating practices, and (5) Emphasizes the impact of sociocultural influences on relationships with food. Subtopics are listed in Table 1. The principles themselves are quite similar to topics covered in more weight-centric nutrition education, illustrating that weight-inclusive nutrition is not a wholesale rejection of nutrition science, but rather an important shift in *how* these nutrition topics are presented.

**Table 1:** Guiding principles of weight-inclusive nutrition.

*Centers a foundation of nutrition science*
Focuses on building understanding of macro and micronutrient function, sources, and recommended intake
*Analyzes the relationship between nutrition and health outcomes*
Counters misinformation about weight and health
Integrates the social determinants of health
Examines the harm stemming from disordered eating and eating disorders
*Examines nutrition information critically*
Teaches skills to find and interpret nutrition information that is reliable, evidence-based, and created by experts
Respects an individual’s own context and knowledge of their body
*Promotes attuned and joyful eating practices*
Emphasizes attunement to hunger and fullness cues
Recognizes the benefits of eating for pleasure and nourishing one’s body with a variety of foods
*Emphasizes the impact of sociocultural influences on relationships with food*
Acknowledges the cultural, systemic, socioeconomic, familial, and peer influences on food choice
Challenges the assumptions of diet culture
Addresses the existence and health impacts of anti-fat bias

The guiding WIN principles were informed by several interdisciplinary public health and social science theories and frameworks. The orientation of the guiding WIN principles aligns with multiple levels of the public health social ecological model (SEM) which emphasizes the multiple factors influencing health behaviors and outcomes, including individual health behaviors, peer and family influences, community-level resources and opportunities as well as societal policies and norms (24). By addressing and naming risk and protective factors across the levels of SEM, public health interventions and policies are more likely to be effective, sustainable, and address systemic inequities that drive health disparities. Through the intentional application of the SEM, the second WIN principle related to nutrition and health centers equity and disrupts narratives that blame individuals by explicitly discussing the social determinants of nutrition and health as well as the multiple influences on nutrition and individual health behavior (25). Bandura’s Social Cognitive Theory (SCT) is a widely utilized theoretical framework in public health and nutrition-related interventions (26) that emphasizes the dynamic interaction between personal factors, behavior, and the environment, which provides a targeted framework for understanding influences on behavior and behavior change (27). Specifically, the nutrition science and nutrition information-related WIN principles correspond with several SCT-related constructs including knowledge and self-efficacy. These principles specifically reflect the SCT “personal factor” domain which underscores the importance of integrating knowledge and skills to develop capacity to sustain behavior through increased self-efficacy (27). Additionally, the WIN principle addressing eating practices emphasizes a skill-based strategy connected to experiential bodily cues and individual knowledge about intuitive eating, further reinforcing self-efficacy by increasing behavioral skills. The WIN principle on sociocultural influences integrates SCT constructs from the social and environmental influences on behavior including “normative beliefs” defined as “cultural norms and beliefs about the social acceptability of a behavior” and social support. Finally, the WIN principles related to nutrition information and nutrition and health draw upon critical health literacy frameworks (28), defined as “the ability to reflect upon health determining factors and processes and to apply the results of the reflection into individual or collective actions for health in any given context.”

### Centers a foundation of nutrition science

Nutrition is a science, and weight-inclusive nutrition should include information about macronutrients and micronutrients. Grounding discussions of nutrition science in terms of dietary nutrients like the macro and micronutrients can facilitate learning about the various nutrients without imparting moral judgments about nutrients. A weight-inclusive approach should discuss the important functions of each nutrient, as well as what foods serve as sources of different nutrients, and when those nutrients may be especially impactful. In this approach, one might consider specific situations when it would be more or less advantageous to eat foods high in a particular nutrient like fat or carbohydrates, allowing individuals to understand that meeting nutrition needs will vary based on a variety of factors. For example, in a sports context, eating a high-fat food might be desirable if completing a long-duration high-energy output activity like a multi-day hike, but may be less beneficial directly before competing in a sprinting event, given the needs of the energy systems involved. This allows for nuance when thinking about how foods high in certain nutrients impact our body functioning rather than dichotomizing foods into broad categories of healthy/unhealthy or good/bad. Information about nutrition science can be tailored to the audience and age group to be most effective (29).

### Analyzes the relationship between nutrition and health outcomes

Many programs, policies, and practices designed to deliver nutrition education discuss the connections between nutrition and health in ways that center weight as the main determinant of health (9). Weight-normative nutrition education will often emphasize the need for “good” nutrition as a means to lose or control weight (30). Weight-inclusive nutrition moves beyond weight to encourage nutrition as a part of health and wellbeing. For example, research shows that when individuals practice health behaviors such as increasing physical activity and decreasing alcohol and tobacco intake, their weight status is not indicative of their health outcomes (12, 31). A recent study by Weeldreyer et al. (32) found that cardiorespiratory fitness predicted cardiovascular disease and all-cause mortality, and mitigated risk associated with weight. Additionally, when discussing nutrition and health, weight-inclusive nutrition emphasizes the social determinants of health, and how our environments, as well as other factors outside our personal control, have large impacts on our health status. Factors like food security, built environment, and exposure to environmental pollutants are emphasized as important contributors to our nutrition status and health outcomes (33–35). Weight-inclusive nutrition-education practices critically examine study design and methodological rigor with the understanding that many studies that find correlations between weight and health outcomes do not account for important potential confounders like health behaviors, experiences of anti-fat bias, internalized anti-fat bias, or weight cycling (9, 12). Furthermore, these studies often do not account for social position factors that intersect with anti-fat bias like socioeconomic status or race and lead to multiple forms of marginalization (36). In this way, weight-inclusive nutrition does not make sweeping conclusions about the connections between weight and health, but rather encourages critical thinking.

Weight-inclusive nutrition also recognizes the importance of teaching about eating-disorder prevention and identification. Although they are inherently nutrition-related, research shows that nutrition educators are uncomfortable and resistant to implementing curricula that address eating disorders (37). Historically, there has been an assumption that talking about disordered eating and eating disorders will somehow cause more harm (38). However, this avoidance perpetuates stigmatization and isolation and is often at odds with what learners need as they explore nutrition through a weight-centric framework. Including eating-disorder education through weight-inclusive nutrition, acknowledges that eating disorders can occur across the weight spectrum (39) and allows individuals to think critically about behaviors that are known to promote disordered-eating and eating-disorder pathology. Ultimately, the inclusion of disordered eating in WIN education may help reduce stigma and provide important skills and language that can serve as a method of eating-disorder prevention (40).

### Examines nutrition information critically

Nutrition information is incredibly prevalent, but finding accurate nutrition information and discerning misinformation is increasingly difficult (41). Weight-inclusive nutrition must address critical nutrition literacy in part because of the prevalence of diet culture and the seductive, yet incorrect information that is often shared widely on social media (42). Critical nutrition literacy is the ability to engage critically with, and reflect upon, how available nutrition information is relevant to individual contexts (43). Much of the information shared on social media reinforces beliefs about food and weight that are weight centric or promote diet culture ideals like the thin/muscular ideals or restrictive diets (44). A weight-inclusive nutrition approach teaches strategies to assess the information found on social media and other forms of media, especially information that promotes restriction or particular body ideals. Furthermore, weight-inclusive nutrition cultivates critical-thinking skills around nutrition research where potential flaws in research design, positionality of the researchers, and conclusions are interrogated. In addition to learning how to carefully examine nutrition information, weight-inclusive nutrition respects an individual’s knowledge of their own body and the context in which they are making food-related decisions. Helping people determine how best to integrate evidence-based nutrition information with their own body knowledge is an important aspect of a weight-inclusive nutrition approach.

### Promotes attuned and joyful eating practices

Instead of focusing on developing a daily “diet” that maintains energy balance which might involve the tracking of calories and encouraging policies like calorie labeling and food restrictions, an inclusive approach to eating practice draws from the principles of intuitive eating (20). Calorie-focused diet plans erase nuances in the metabolic impacts of different nutrients, ignore an individual’s own preferences, encourage feelings of guilt and shame when restriction does not result in weight loss, and lead to increasingly disordered eating behaviors (45, 46). Conversely, intuitive eating, one aspect of weight-inclusive nutrition, emphasizes the need to recognize and listen to your own body’s cues around hunger, fullness, and satisfaction. Intuitive eating is not just the absence of dieting behaviors, but a sense of attunement with your own body’s needs and eating for satisfaction. This is especially important when hunger and fullness cues have been muted as a result of chronic, long-term dieting (47). Although nutrition is still important in an intuitive-eating framework, satisfying nutrient needs is not the sole purpose of eating, as eating for enjoyment, social reasons, or in response to emotions are all recognized as valid. Intuitive eating also emphasizes the need to give yourself unconditional permission to eat, meaning that all desired foods can fit into one’s daily meals which helps to reduce the tendency to dichotomize food into categories that are allowed or restricted, a mental pattern that can lead to increased anxiety around food (48). While respecting that food can be one coping mechanism for emotions, a weight-inclusive nutrition education approach recognizes that eating in response to emotions or feeling anxiety around food can indicate a need for additional mental health coping mechanisms and support (49). Mental health is greatly impacted by one’s relationship with food (50), and weight-inclusive approaches honor the impact both mental health and dietary patterns have on overall wellbeing. The benefits of taking an intuitive-eating approach include improved diet quality, physical health, and physiological health markers (51–53). With weight-inclusive nutrition, eating in practice is flexible, individual, responsive to different daily needs, and not a source of negative emotions. In fact, by removing food restrictions and empowering individuals to eat in a way that feels best to them, weight-inclusive nutrition encourages people to celebrate foods they love and find joy in eating (54).

### Emphasizes the impact of sociocultural influences on relationships with food

The social-ecological model identifies several layers of influence on an individual’s health choices and outcomes, such as policies, community and societal norms (i.e., social media), organizational influence (i.e., schools and workplaces), interpersonal influences (i.e., friends and family), and individual knowledge and attitudes (24). Using a weight-inclusive approach to nutrition, acknowledging the role that these sociocultural influences have on individual food choice (55–57) is a key factor when discussing nutrition and health outcomes. These influences all exist within the wider context of diet culture and anti-fat bias that permeate Western culture, constructing a moral hierarchy of bodies (58).

As mentioned earlier, nutrition policies and public health campaigns aimed at weight loss (i.e., campaigns to reduce “childhood obesity”) falsely conflate weight and health (12) and may lead individuals to choose foods that facilitate weight loss rather than foods that provide adequate nutrition as well as satisfaction and joy. Diet culture and anti-fat bias are embedded in the formal and informal education that individuals receive about nutrition, including in schools and workplaces (30, 59). Adults may be encouraged to engage in workplace weight loss challenges, implying that weight loss, rather than improved health behaviors, should be rewarded (60). Children may be taught explicitly in formal health class to avoid certain foods that are “bad” for them because those foods would cause them to gain weight (61). Similarly, family dynamics and peers play a complex role in teaching about food choice (57). For instance, peers may tease each other about their weight, or parents may restrict certain foods, like processed foods, from their children’s diets. Friends and family also model eating behaviors, and young people especially notice how others around them are behaving around food (62). It becomes difficult to model, for example, the potential benefits of carbohydrate intake to a child if a guardian of that child is scared to consume carbohydrates for fear of gaining weight. Diet culture can also be seen in well-meaning social movements like the push for “sustainable” eating, where people are encouraged to consume or avoid certain foods to promote environmental outcomes. Although well-intentioned, pushing individuals to adopt “sustainable” diets may, in reality, function as a set of restrictive rules, and shift blame for environmental degradation from public policy choices to individual food choices (63). Therefore, discussing nutrition from a weight-inclusive perspective requires utilizing a critical lens to dissect the impact that diet culture and anti-fat bias have on structural policies as well as interpersonal relationships, and subsequently, food choice and diet-related behaviors. In contrast to the negative impacts that diet culture and anti-fat bias may have on people’s food choices, weight-inclusive nutrition celebrates the ways that food can build community, enhance a sense of identity, and play a central role in celebrations in many cultures (64).

## Discussion

Individuals and society are often indirectly educated about nutrition through research, policy, and practice. While the tenets discussed above consider curriculum integration and foundational knowledge often found in schools, a weight-inclusive nutrition perspective can be applied in both formal and informal education contexts. Similar to other aspects of health and wellbeing, the weight-inclusive nutrition principles defined in this paper exist outside of the classroom.

Formal education contexts could include nutrition curricula for K-12 schools, college and postsecondary education for future healthcare professionals such as dietitians, and extend to continued education opportunities for educators and health professionals to improve comprehensive and patient-centered care. Nutrition education will also occur in informal ways across settings such as clinical dietetics, athletics, and public-health campaigns. Table 2 outlines opportunities in which to formally and informally apply weight-inclusive nutrition education. Approaching research from a weight-inclusive perspective can help disentangle the true relationships between weight and health while appropriately taking into account experiences of anti-fat bias, social identities, and demographic factors. Individuals would then have more accurate information with which to make personal nutrition choices. Approaching policies from a weight-inclusive perspective can help the public better understand how to integrate internal body cues with basic nutrition information rather than relying on external cues (like calorie counting) or engaging in disordered, restrictive behaviors. Further, weight-inclusive nutrition practices could help clients and patients develop a positive relationship to food and body and prevent dangerous eating-disorder pathology.

**Table 2:** Examples of weight-inclusive nutrition in formal and informal educational contexts.

Context	Example application(s)
Formal nutrition education contexts	
Health education in K-12 schools	Teaching the pitfalls of the BMI Discussing the benefits of health behaviors Practicing how to recognize hunger and fullness cues Focusing on how different nutrients impact our health Celebrating the joy, social connection, and cultural meaning that food can bring to our lives
Courses aimed at future nutrition and dietetics professionals	Teaching basic research literacy, so practitioners can critically assess nutrition research Understanding the principles of intuitive eating and how to integrate them into practice Recognizing the impacts of social determinants of health on client’s nutrition choices Screening for eating disorders and practicing a trauma-informed approach
Courses aimed at other healthcare providers	Illuminating the impact of medical anti-fat bias on patient’s overall health as well as nutrition intake Deconstructing common fad diets that are often recommended to patients Providing basic education about nutrition science, so healthcare providers are more informed about macro and micronutrients
Informal nutrition education contexts	
Nutrition policy	Integrating weight-based bullying into anti-discrimination and harassment policies at workplaces and schools Advocating for weight discrimination to be included as a category of illegal discrimination Examining federal nutrition policies to emphasize the need to establish healthy relationships with food, not to eat to achieve weight goals Rethinking the value, goal, and efficacy of policies like menu calorie labeling, front of package labeling, or BMI report cards Allowing students to have adequate time in school to eat and respond to their hunger/fullness cues
Athletics	Teaching athletes basic sports nutrition principles Emphasizing the performance detriments of relative energy deficiency, and the need to fuel appropriately for sport performance
Public health campaigns	Reenvisioning “obesity prevention” campaigns to focus not on weight but on health behaviors with a goal of health promotion rather than weight loss or control Advocating for equal access to all foods, and an end to food insecurity Focusing on educational campaigns that provide positive nutrition messaging rather than fearmongering around particular nutrients

Weight-inclusive nutrition allows learners to understand that health and well-being occurs for people across the weight spectrum, that internal, rather than external cues, can be utilized to foster a healthy relationship to food and body, and that disordered eating and eating disorders do occur and should be acknowledged. This framework creates more inclusive nutrition education without replacing the traditional principles that are foundational to the understanding of nutrition science. Rather, they enhance the way nutrition science is understood, practiced, and internalized for those across the weight and size spectrums without limiting wellbeing or perpetuating eating disorder pathology.

## Data Availability

The original contributions presented in the study are included in the article/supplementary material, further inquiries can be directed to the corresponding author.
